# Similarity score for screening phase-retrieved maps in X-ray diffraction imaging – characterization in reciprocal space

**DOI:** 10.1107/S1600577523009827

**Published:** 2024-01-01

**Authors:** Yuki Takayama, Masayoshi Nakasako

**Affiliations:** a Graduate School of Science, University of Hyogo, 3-2-1 Kouto, Kamigori-cho, Ako-gun, Hyogo 678-1297, Japan; b RIKEN SPring-8 Center, 1-1-1 Kouto, Sayo, Sayo-gun, Hyogo 679-5148, Japan; c Synchrotron Radiation Research Center, Hyogo Science and Technology Association, 1-490-2 Kouto, Shingu, Tatsuno, Hyogo 679-5148, Japan; d International Center for Synchrotron Radiation Innovation Smart, Tohoku University, Katahira 2-1-1, Aoba-ku, Sendai 980-8577, Japan; e Department of Physics, Faculty of Science and Technology, Keio University, 3-14-1 Hiyoshi, Kohoku-ku, Yokohama, Kanagawa 223-8522, Japan; RIKEN SPring-8 Center, Japan

**Keywords:** X-ray diffraction imaging, phase retrieval calculation, phase problem

## Abstract

X-ray diffraction imaging (XDI) is utilized for visualizing the structures of non-crystalline particles in material sciences and biology. In the structural analysis, phase-retrieval (PR) algorithms are applied to the diffraction amplitude data alone to reconstruct the electron density map of a specimen particle projected along the direction of the incident X-rays. However, PR calculations may not lead to good convergence because of a lack of diffraction patterns in small-angle regions and Poisson noise in X-ray detection. Therefore, the PR calculation is still a bottleneck for the efficient application of XDI in the structural analyses of non-crystalline particles. For screening maps from hundreds of trial PR calculations, we have been using a score and measuring the similarity between a pair of retrieved maps. Empirically, probable maps approximating the particle structures gave a score smaller than a threshold value, but the reasons for the effectiveness of the score are still unclear. In this study, the score is characterized in terms of the phase differences between the structure factors of the retrieved maps, the usefulness of the score in screening the maps retrieved from experimental diffraction patterns is demonstrated, and the effective resolution of similarity-score-selected maps is discussed.

## Abstract 2

The similarity score is a useful metric for screening electron density maps from phase-retrieval calculations in X-ray diffraction imaging. The characteristics of the score have been studied in reciprocal space and are described here.

## 1. Introduction

X-ray diffraction imaging (XDI) is a technique for visualizing the structures of non-crystalline particles of size in the micro- to sub-micrometre range (Miao *et al.*, 1999 ▸, 2015 ▸; Nakasako, 2018 ▸; Nakasako *et al.*, 2020 ▸). In an XDI experiment, the particle to be imaged is irradiated by an X-ray beam with almost complete spatial coherence, and the Fraunhofer diffraction pattern of the particle is recorded at a high sampling frequency. When the sampling frequency satisfies the oversampling condition (Miao *et al.*, 2003*a* ▸), the electron density map of the particle projected along the direction of the incident X-ray beam is, in principle, reconstructed from the oversampled diffraction amplitudes alone using phase-retrieval (PR) algorithms (Fienup, 1978 ▸, 1982 ▸).

Owing to the penetration power of short-wavelength X-rays, XDI is advantageous for visualizing whole structures of non-crystalline particles without sectioning and chemical labeling. Therefore, XDI has been applied to structural analyses of non-crystalline particles in material sciences and biology by using synchrotron radiation (SR) X-rays (Williams *et al.*, 2003 ▸; Shapiro *et al.*, 2005 ▸; Miao *et al.*, 2006 ▸; Nishino *et al.*, 2009 ▸; Jiang *et al.*, 2010 ▸; Takayama & Nakasako, 2012 ▸; Nam *et al.*, 2013 ▸; Takayama *et al.*, 2018 ▸; Kobayashi *et al.*, 2018*a* ▸) and X-ray free-electron laser (XFEL) pulses (Seibert *et al.*, 2011 ▸; Loh *et al.*, 2012 ▸; Nakasako *et al.*, 2013 ▸; Takahashi *et al.*, 2013 ▸; Gallagher-Jones *et al.*, 2014 ▸; Hantke *et al.*, 2014 ▸; Xu *et al.*, 2014 ▸; Kimura *et al.*, 2014 ▸; Oroguchi *et al.*, 2015 ▸; Takayama *et al.*, 2015*a* ▸; van der Schot *et al.*, 2015 ▸; Ekeberg *et al.*, 2015 ▸; Kobayashi *et al.*, 2016*a* ▸; Kameda *et al.*, 2017 ▸; Oroguchi *et al.*, 2018 ▸; Nakasako, 2018 ▸; Nakasako *et al.*, 2020 ▸; Ayyer *et al.*, 2021 ▸; Cho *et al.*, 2021 ▸; Kobayashi *et al.*, 2021 ▸; Uezu *et al.*, 2023 ▸). The resolutions of the structure analyses were several tens of nanometres and sometimes reached several nanometres.

When the diffraction pattern of a particle is recorded without any pattern loss or noise, the projected electron density map of the particle can be, in principle, retrieved (Barakat & Newsam, 1984 ▸). However, PR calculations for experimental diffraction patterns frequently give non-realistic maps due to the loss of small-angle regions hidden by the beamstop and Poisson noise in X-ray detection (Huang *et al.*, 2010 ▸; Kobayashi *et al.*, 2014 ▸; Takayama *et al.*, 2015*b* ▸; Sekiguchi *et al.*, 2016 ▸, 2017 ▸). In particular, in our experiences the loss of larger small-angle regions makes the convergence of PR calculations to realistic maps more difficult (Kobayashi *et al.*, 2014 ▸), because the small-angle regions contain structural information on the overall shape and the total electrons of the particle.

**Figure 1:**
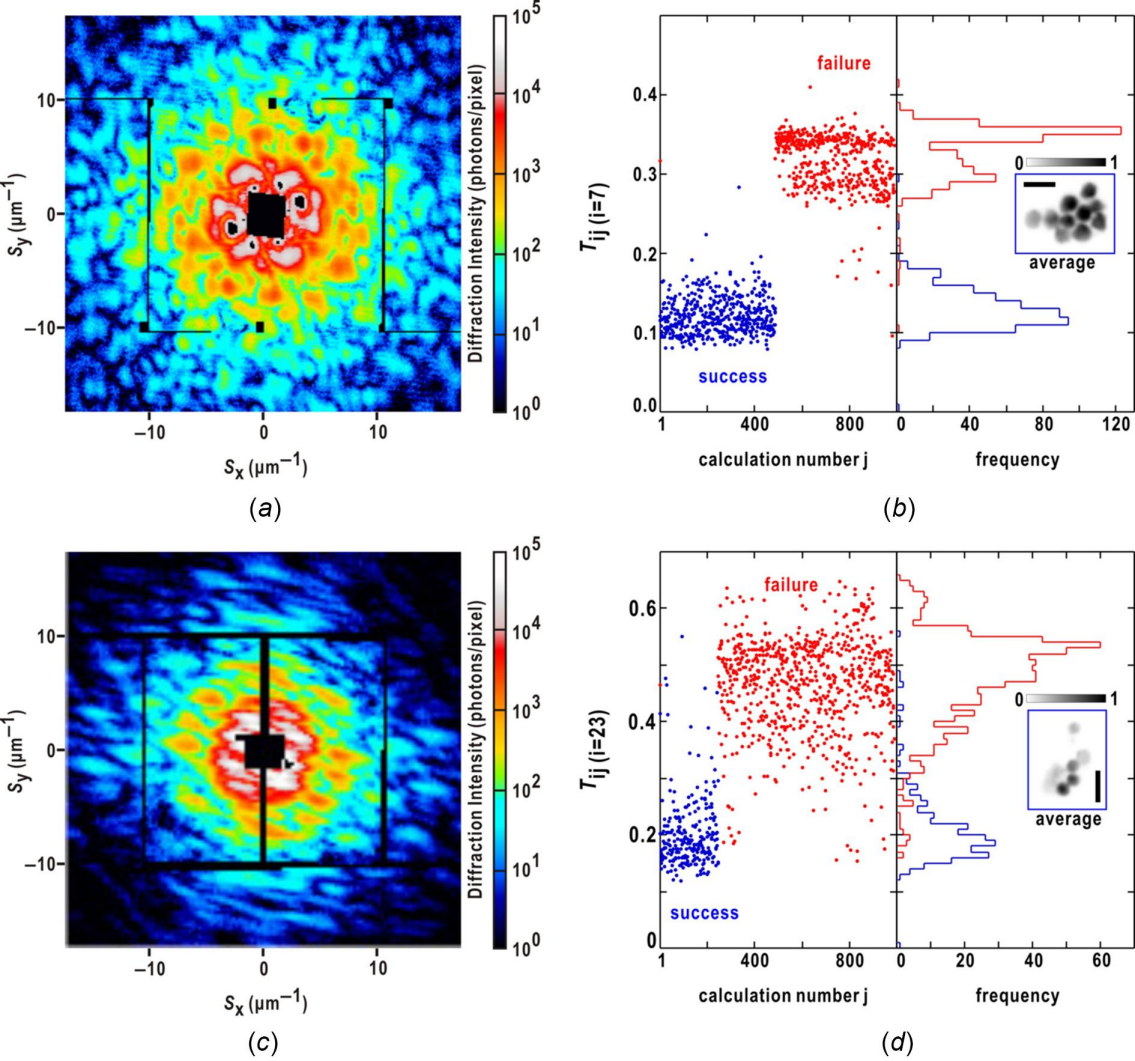
(*a*) Single-shot diffraction pattern from an aggregate of ten colloidal gold particles with 250 nm diameter (Sekiguchi *et al.*, 2016 ▸, 2017 ▸). The black square at the center is the shadow of the beamstop, and the stripes in the horizontal and vertical directions are the gaps between the CCD panels of the detectors. (*b*) The similarity scores of 1000 retrieved maps against the reference (left plot), and the frequency distribution of the scores of 999 maps (right). The reference map was obtained from the seventh trial (*i* = 7) of the 1000 PR calculations. The symbols and lines for correct and incorrect maps are colored in blue and red, respectively. The inset shows electron density maps averaged over the correct maps. (*c*) Single-shot diffraction pattern from an aggregate of colloidal gold particles. (*d*) Distribution of the similarity scores of 1000 maps retrieved from the diffraction pattern in panel (*c*). The reference map was obtained from the 23rd trial (*i* = 23) of the 1000 PR calculations. In panels (*b*) and (*d*), calculation trials were numbered after manual classifications judging whether the final maps were realistic. Panels (*a*) and (*c*) are reused from our previous paper (Sekiguchi *et al.*, 2017 ▸) after modification with permission from International Union of Crystallography.

As typical examples, we show PR calculations for single-shot diffraction patterns from clusters of colloidal gold particles in Fig. 1 ▸ (Sekiguchi *et al.*, 2017 ▸). In the diffraction pattern in Fig. 1 ▸(*a*), the area of the lost small-angle region was more than three times the reciprocal of the cluster size, and the PR calculations yielded realistic maps with approximate probabilities of 50% [Fig. 1 ▸(*b*)]. The diffraction pattern of Fig. 1 ▸(*c*) loses the small-angle region as well as the pattern in Fig. 1 ▸(*a*), and signal-to-noise ratios beyond 10 µm^−1^ were smaller than those in Fig. 1 ▸(*a*). As a result, the probability yielding realistic maps was approximately 30% [Fig. 1 ▸(*d*)].

Therefore, to efficiently perform XDI structural analysis, protocols and/or metrics are necessary for screening the retrieved maps from a number of independently performed PR calculations (Chen *et al.*, 2007 ▸; Martin *et al.*, 2012 ▸; Park *et al.*, 2013 ▸; Rodriguez *et al.*, 2013 ▸; Kobayashi *et al.*, 2014 ▸; van der Schot *et al.*, 2015 ▸; Ekeberg *et al.*, 2015 ▸; Sekiguchi *et al.*, 2016 ▸; Favre-Nicolin *et al.*, 2020 ▸). For screening maps (Sekiguchi *et al.*, 2017 ▸), we propose the use of the similarity score (Miao *et al.*, 2003*b* ▸), defined as

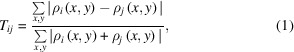

where ρ_*i*_(*x*, *y*) denotes the projected electron density of the *i*th map. As XDI theory is formulated based on the first Born-approximation on a weak-phase object, ρ_*i*_(*x*, *y*) actually represents the projected electron density and not the complex transmission function formulated in ptychography (Pfeiffer, 2018 ▸).

Our previous structural analyses on the aggregates of colloidal metal particles (Sekiguchi *et al.*, 2017 ▸; Oroguchi *et al.*, 2018 ▸) showed that probable images were obtained with a high probability when each pair of the retrieved maps among a set of independently retrieved maps displayed similarity scores smaller than a threshold value (0.2) (Sekiguchi *et al.*, 2017 ▸) (Fig. 1 ▸). In contrast, when PR calculations failed, the resultant maps were inconsistent with each other and yielded similarity scores greater than the threshold value. The threshold value acts as a necessary condition to extract probable maps, although it is insufficient for the purpose because the score becomes smaller than the threshold value even for pairs of failed maps with similar structures. Owing to the success and usefulness in screening out non-realistic maps, we have been using the similarity score for XDI structural analyses (Kobayashi *et al.*, 2016*a* ▸,*b* ▸; Sekiguchi *et al.*, 2017 ▸; Oroguchi *et al.*, 2018 ▸; Nakasako *et al.*, 2020 ▸; Kobayashi *et al.*, 2021 ▸). However, it is unclear as to why the similarity score works as a good metric for screening retrieved maps.

In this study, we investigate how the similarity score works in the screening of retrieved maps in XDI. We describe equation (1) in reciprocal space to understand the correlation between the quality of the retrieved maps, and characterize the similarity score in terms of phase differences between the structure factors of the maps. Through screening the maps retrieved from diffraction patterns obtained in XFEL-XDI and SR-XDI experiments, we assess the usefulness of the score and the resolution-dependent contributions of phase differences to the score.

## 2. Theoretical background

### 2.1. Interpretation of the similarity score in reciprocal space

#### 2.1.1. Expression of the similarity score by Fourier transform

To characterize the similarity score in reciprocal space, we express the *i*th projection map, ρ_*i*_(*x*, *y*) in equation (1), as the inverse Fourier transform of the structure factor, *F*_*i*_(**S**), where **S** is the scattering vector. Equation (1) in reciprocal space can be written as

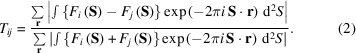

According to the PR algorithm (Fienup, 1982 ▸), the final map is given by the inverse Fourier transform of the structure factor composed of the observed structure amplitude, |*F*_obs_(**S**)|, and the phase in the final calculation cycle, α_*i*_(**S**). Therefore, equation (2) is modified as

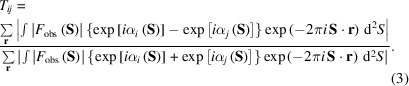

Equation (3) indicates that the similarity score depends on the phase differences between the structure factors of the two maps.

**Figure 2:**
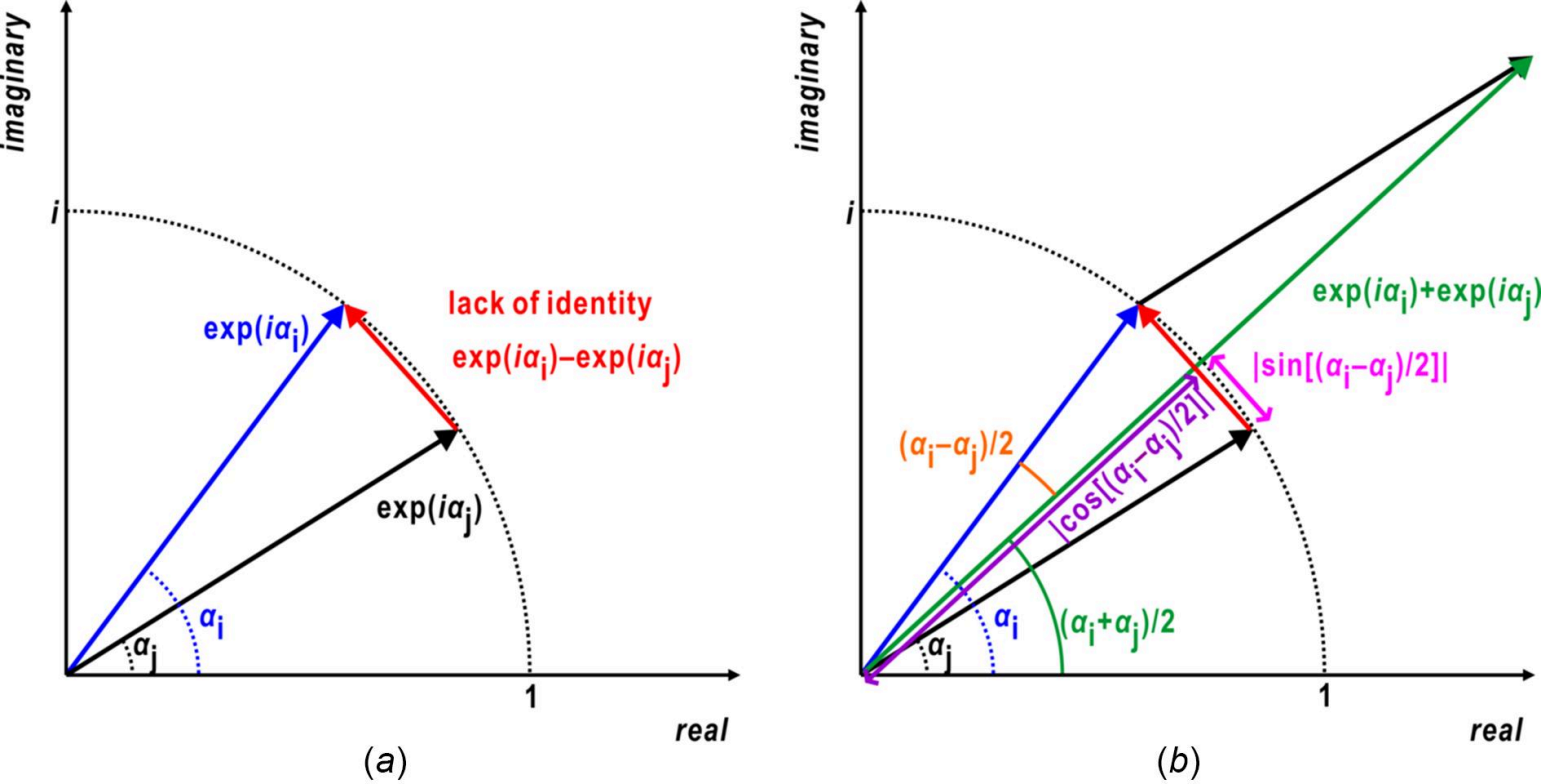
(*a*) Definition of the LoI in an Argand diagram. (*b*) Diagram showing the phase terms appearing in equation (4).

We designate the term 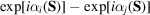 as the ‘lack of identity’ (LoI) [Fig. 2 ▸(*a*)], analogous to the lack of closure of structure factors in the multiple isomorphous replacement method (Blow & Crick, 1959 ▸). Using the following relation on the difference and sum of the phase terms [Fig. 2 ▸(*b*)],

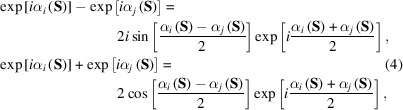

then equation (3) is modified as

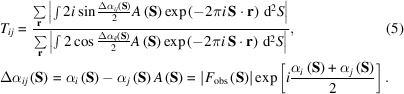

The trigonometric terms of Δα_*ij*_(**S**) originating from the LoI act as weights on the structure factor *A*(**S**), which is a product of |*F*_obs_(**S**)| and the phase averaged between the structure factors of the *i*th and *j*th maps. As the structure amplitudes at the lower resolution are greater than those at the higher resolution, the phase differences of the structure factors at the lower resolution dominate the score. With respect to two similar maps, the phase difference of the structure factors at low resolution will be as small as zero and reduce the numerator.

#### 2.1.2. Resolution-dependent variation of the Fourier term

As described above, the trigonometric terms of Δα_*ij*_(**S**) and the structure amplitudes vary depending on the resolution. This implies that the similarity score can display a correlation with the resolution of the pair of maps, although the similarity score does not obviously depend on the resolution. Thus, it is meaningful if we modify each of the denominator and numerator in equation (5) into formulae suitable for monitoring the resolution-dependent variations of the trigonometric term. By using 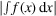 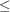 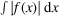 , the numerator and denominator in equation (5) satisfy the following inequalities,

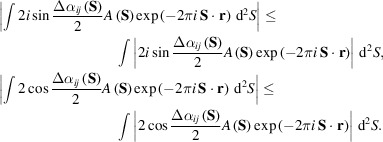

Then, using the equation |*f*(*x*)*g*(*x*)| = |*f*(*x*)||*g*(*x*)|, the right-hand-side term in each of the above inequalities is expressed as the sum of the integrals on the *N*-divided resolution shells and each integrand is separated as

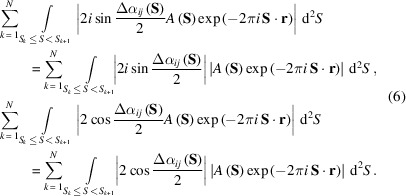

Therefore, the separated trigonometric term may be helpful for numerically evaluating the influence of each resolution-shell to the similarity score.

#### 2.1.3. Influences of phase differences in the resolution shell

Based on equation (6), to roughly estimate the influences of the phase differences in each resolution shell we use the following two quantities averaged over the maps in the narrow *k*th resolution shell,

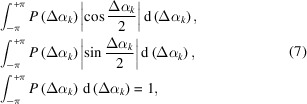

where *P*(Δα_*k*_) is the normalized appearance frequency of Δα_*k*_. Hereafter, we designate the first and second equations as averaged cosine and sine terms in the *k*th shell, respectively.

Using equation (7), the influence of the trigonometric terms is qualitatively evaluated. For maps similar to each other, small phase differences between the structure factors in the low-resolution shell will yield values of the averaged cosine and sine term as large as 1 and 0, respectively. In contrast, for high-resolution shells, where the ambiguity of structure amplitudes due to Poisson noise causes large phase differences between the two structure factors, the averaged cosine term decreases, while the averaged sine term increases.

### 2.2. Reference values from special cases

As equation (6) is complicated, here we show values of the averaged cosine and sine terms in two special cases, which may be useful as references for roughly estimating the influences of the phase differences on the similarity score.

In the first special case, we assume that a particle has a circular shape with radius *r*_0_ and uniform density. Then, the structure factor is written by the following equation (Born & Wolf, 2001 ▸),

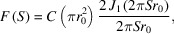

where *J*_1_ is the first-order cylindrical Bessel function and *C* is a constant. The phase value between any pair of adjacent zero-crossing points of *J*_1_ is zero or π. When PR calculations for the diffraction amplitudes give two maps of circular shapes with different radius, the phase difference values are zero or π in any narrow resolution shell. Therefore, the phase differences between the two maps in the *k*th resolution shell are a constant.

In such a case, without the two inequalities introduced for the general case described above, the similarity score in the *k*th resolution shell is simplified as

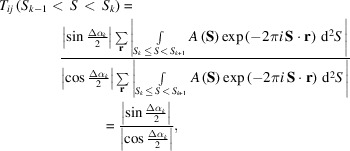

where Δα_*k*_ is a constant phase-difference value in the *k*th resolution shell. When Δα_*k*_ = 0, the sine and cosine terms are 0 and 1, respectively, resulting in the similarity score in the resolution shell becoming 0. In contrast, when Δα_*k*_ = π, the maximum phase difference, then the sine and cosine terms are 1 and 0, and the score diverges to infinity.

In this regard, we express an electron density distribution using the multipole expansion (Stuhrmann & Miller, 1978 ▸). Then, the electron density distribution is the sum of the first monopole term, the second dipole term, the third quadrupole and higher-order terms. If the weight for the first monopole term is approximated as uniform, the structure factor has the formula for a circular shape described above. In addition, when the monopole term approximating the size of the density distribution has a large electron density contrast (Ibel & Stuhrmann, 1975 ▸), the Fourier transform of the monopole term is dominant in the structure factor of the density distribution in the small-angle region. Therefore, this simple numerical calculation may be helpful to roughly evaluate the influences of the phase-difference terms in equation (6) on the similarity score.

In the next special case, we numerically calculate the averaged cosine and sine terms for the case that the frequency distribution of phase differences is random among the structure factors of retrieved maps in the *k*th resolution shell. Then, the values of the averaged cosine and sine terms approach 0.64 as

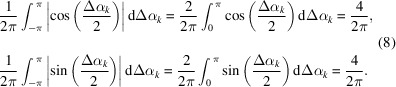

Hereafter, this value is designated the random phase limit.

### 2.3. Figure of merit for estimated phase

In X-ray crystallography and transmission electron microscopy (TEM) of biological macromolecules, the reliability of a reconstructed map is evaluated using the figure of merit (FoM) (Blow & Crick, 1959 ▸), based on the probability distribution of phase values in the experimental estimation, as follows,

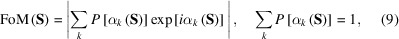

where *P*[α_*k*_(**S**)] is the probability of the *k*th bin of the phase values at the scattering vector **S**. The reconstructed maps displaying a FoM greater than the threshold of 0.5 are regarded as interpretable (Lunin & Woolfson, 1993 ▸; Perrakis *et al.*, 1997 ▸). The threshold indicates that the phase values are distributed within ±60° from the true value (Drenth, 1994 ▸). The FoM for the structure factors of two maps is in correlation with the LoI as

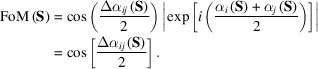

Thus, the similarity-score-selected maps were evaluated using FoM and LoI.

### 2.4. Phase-retrieval transfer function for estimated phase

In XDI structural analyses, the phase retrieval transfer function (PRTF) is frequently used to estimate the effective resolution for a set of retrieved maps (Chapman *et al.*, 2006 ▸). The PRTF for a set of retrieved maps is defined as

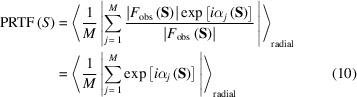

where *M* is the number of retrieved maps incorporated into the calculation, and 〈…〉_radial_ refers to circular averaging with respect to the scattering vector length. The effective resolution is determined from the reciprocal of the scattering vector length, where the PRTF curve decreased to a specified threshold value. The PRTF for the structure factors of the two retrieved maps is derived using equation (4) as

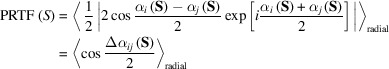

where we use the fact that the cosine term is positive for −π ≤ Δα_*ij*_(**S**) ≤ +π. The effective resolution estimated using the PRTF will be discussed in conjunction with the similarity score in the *Discussion* section[Sec sec5].

## 3. Experimental procedure and structure analysis

### 3.1. Specimen preparation

For XFEL-XDI experiments, colloidal gold particles with a mean diameter of 250 nm (BBI Solutions, UK) were dispersed on custom-made specimen disks, each of which had a 1 mm × 1 mm Si_3_N_4_ membrane window of 100 nm thickness (Norcada, Canada). Further details were described in our previous publications (Sekiguchi *et al.*, 2014*a* ▸, 2016 ▸; Kobayashi *et al.*, 2016*a* ▸,*b* ▸; Nakasako, 2018 ▸; Nakasako *et al.*, 2020 ▸).

For the SR-XDI experiments, we used commercially available specimen disks with a 5 mm × 5 mm Si_3_N_4_ membrane window (Silson Ltd, UK). The windows were coated with ∼15 nm-thick carbon layers using a JEE-420 vacuum evaporator (Jeol, Japan) and then covered with poly l-lysine layers (Sigma-Aldrich, USA) to assist the adhesion of the specimen particles to the Si_3_N_4_ membranes (Takayama & Yonekura, 2016 ▸; Kobayashi *et al.*, 2016*b* ▸). We prepared specimen disks adsorbing colloidal gold particles with a mean diameter of 400 nm (Sigma-Aldrich, USA) or gold urchin particles synthesized according to the literature (Wang *et al.*, 2012 ▸).

### 3.2. XFEL-XDI experiments and data processing

XFEL-XDI experiments were performed using our custom-made apparatus KOTOBUKI-1 (Nakasako *et al.*, 2013 ▸) at beamline BL3 of SACLA (Tono *et al.*, 2013 ▸). Focused XFEL pulses with a photon energy of approximately 5.5 keV (corresponding to a wavelength of 0.225 nm) had intensities of 10^10^–10^11^ photons pulse^−1^ µm^−2^ and almost complete spatial coherence (Kobayashi *et al.*, 2018*a* ▸). The specimen mounted on the goniometer of KOTOBUKI-1 was scanned with a 50 µm step against the incident XFEL pulses, and single-shot diffraction patterns were recorded using multi-port CCD (MPCCD) octal and dual detectors (Kameshima *et al.*, 2014 ▸). After each scan, the recorded diffraction patterns were processed using the *G-SITENNO* program suite (Sekiguchi *et al.*, 2014*a* ▸,*b* ▸), which extracted diffraction patterns from a cluster of colloidal gold particles with a signal-to-noise ratio greater than 2 at a resolution of 15 µm^−1^. Further details of the experimental setup, procedures and data processing were reported previously (Sekiguchi *et al.*, 2014*a* ▸, 2016 ▸; Kobayashi *et al.*, 2016*a* ▸,*b* ▸; Nakasako, 2018 ▸; Nakasako *et al.*, 2020 ▸; Kobayashi *et al.*, 2021 ▸; Uezu *et al.*, 2023 ▸).

### 3.3. XDI experiments using synchrotron X-rays and data processing

The SR-XDI experiments were performed using an originally developed atmospheric XDI system (Takayama *et al.*, 2018 ▸, 2021 ▸) installed at the imaging station of BL24XU in SPring-8 (Takayama *et al.*, 2020 ▸). Specimen particles were irradiated with the nearly plane-wave region of the incident X-rays produced using a 30 µm pinhole, which was placed approximately 1.5 m upstream of the specimen position.

An aggregate of gold urchin particles was irradiated by X-rays with a photon energy of 8.000 keV (corresponding to a wavelength of 0.1550 nm), and the diffraction pattern was recorded using a PILATUS 100k detector (Dectris Ltd, Switzerland) (Kraft *et al.*, 2009 ▸) placed approximately 4.1 m downstream of the specimen. An aggregate of 400 nm colloidal gold particles was irradiated by X-rays with a photon energy of 8.310 keV (corresponding to a wavelength of 0.1492 nm), and the diffraction pattern was recorded using an EIGER X 1M detector (Dectris Ltd, Switzerland) (Dinapoli *et al.*, 2011 ▸) placed approximately 3.2 m downstream of the specimen.

For each specimen, a background pattern was recorded from an area of the Si_3_N_4_ membrane yielding no speckles, and was subtracted from the diffraction pattern of the specimen. Centro-symmetric averaging was applied to the background-subtracted diffraction pattern to improve the signal-to-noise ratio owing to good Friedel symmetry.

In the SR-XDI experiments described above, as the coherent flux of SR X-rays from the undulator of BL24XU was around 0.1% due to the size of the light source (electron bunch) in the SPring-8 storage ring (approximately 300 µm along the horizontal direction), we extracted the coherent part of the incident X-rays using a pinhole with a diameter of 30 µm, and a specimen was irradiated by the plane-wave part at the peak area of the diffraction from the pinhole (Takayama *et al.*, 2018 ▸). In this case, the background scattering predominantly came from the Si_3_N_4_ membrane irradiated by the foot region of the pinhole-diffracted X-rays, where the spatial coherence was low. The electron density in the membrane was assumed to be uniform at the maximum resolution of the experiment. In addition, the background scattering from the membrane was assumed to be incoherent and the interference between the background scattering and the diffraction wave from the specimen was negligible. Therefore, the background subtraction procedure was routinely applied as reported for SR-XDI experiments at BL24XU (Takayama *et al.*, 2018 ▸) and BL29XUL (Nishino *et al.*, 2009 ▸; Nam *et al.*, 2013 ▸; Kobayashi *et al.*, 2018*b* ▸) at SPring-8 and also for XDI experiments at BL9C at Pohang Light Source II (Ahn *et al.*, 2021 ▸).

### 3.4. PR calculation and multivariate analysis

We performed PR calculations for the diffraction patterns using a custom-made Python3 software, in which the hybrid-input–output (HIO) (Fienup, 1982 ▸), shrink-wrap (SW) (Marchesini *et al.*, 2003 ▸) and error reduction (ER) algorithms (Gerchberg & Saxton, 1972 ▸) were implemented. Each of 1000 independent PR calculations started with a random-valued map different among the 1000 calculations. An initial support was set as the area where the magnitude of the real-space autocorrelation function calculated from the diffraction pattern was greater than 4% of the maximum. Each projected electron density map was retrieved by 10000 iterations of the HIO cycles with SW modifications at every 100 HIO-cycles and additional 1000 iterations of the ER calculation. Details of HIO and SW were reported previously (Kodama & Nakasako, 2011 ▸; Oroguchi & Nakasako, 2013 ▸).

Based on the assumption that realistic maps were obtained at high frequency and similar to each other when a PR calculation is successful, we searched a reference map for a calculation of the similarity score among the set of retrieved maps as follows. The 1000 retrieved maps were first translated to coincide the center of gravity of the density with that of an arbitrarily selected reference map, and we calculated the similarity score for whole combinations of 1000 maps (499500 pairs). Then, we selected the pair of maps yielding the smallest similarity score as the most probable pair, and one of the pair was used as a reference map for the subsequent calculation. Each map was translated to maximize phase-only correlation (Kuglin & Hines, 1975 ▸) against the reference map. In the PR calculation, several maps in the π-rotation relative to the reference map appeared owing to the centrosymmetry of the diffraction patterns. These maps were corrected with respect to π-rotation during translational alignment using the phase-only correlation. After the correction, the similarity scores for whole combinations of 1000 maps were again calculated against the reference. It should be noted that either of the map pair gave almost the same result of the calculated similarity scores.

Independently, the maps were classified into ten groups by *K*-means clustering (MacQueen, 1967 ▸) without the information from the similarity score. When assuming that the maps are classified into ten groups, we minimize the sum of squared distances between the maps and the centroids of the groups defined as

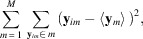

where **y**_*im*_ is a vector indicating the position of the *i*th map belonging to the *m*th group and 〈**y**_*m*_〉 is the centroid of the *m*th group. For visualizing the distributions of the classified ten groups, the map positions were projected onto a plane spanned by the two principal component (PC) vectors, which had the largest and second largest eigenvalues in the principal component analysis (PCA) (Jolliffe & Cadima, 2016 ▸) applied to the 1000 retrieved maps. It should be noted that, on the plane spanned by the first and second PC vectors, clusters separated along the third-order or higher-order vectors will be overlayed because the *K*-means clustering was applied before the dimensionality reduction by PCA. Details of the PCA for a number of PR maps were reported previously (Sekiguchi *et al.*, 2016 ▸). The *scikit-learn* library (Pedregosa *et al.*, 2011 ▸) was used for the *K*-means clustering and PCA.

Finally, in each class, the phase distributions among the structure factors of the maps were evaluated using the FoM at every scattering vector [equation (9)] (Sekiguchi *et al.*, 2017 ▸), and the phase differences in the structure factors between any pair of maps were represented using the averaged cosine and sine terms of equation (7) in each resolution shell.

## 4. Results

Here, we performed the screening of maps phase-retrieved from experimental diffraction patterns using the similarity score in real space and interpreted the score in terms of phase differences between the structure factors of the retrieved maps. In the analyses, we targeted diffraction patterns from aggregates of colloidal gold particles, because the known size and shape of the particles made identifying realistic maps easy.

### 4.1. Application to single-shot diffraction patterns recorded using XFEL pulses

Here, we assessed the similarity score for the maps retrieved from single-shot diffraction patterns recorded using XFEL pulses (Fig. 1 ▸). As a lot of single-shot diffraction patterns can be collected within a short time in our XFEL-XDI experiments (Kobayashi *et al.*, 2016*a* ▸; Nakasako, 2018 ▸), efficient and automatic screening of probable and non-realistic maps is necessary in the structure analyses.

**Figure 3:**
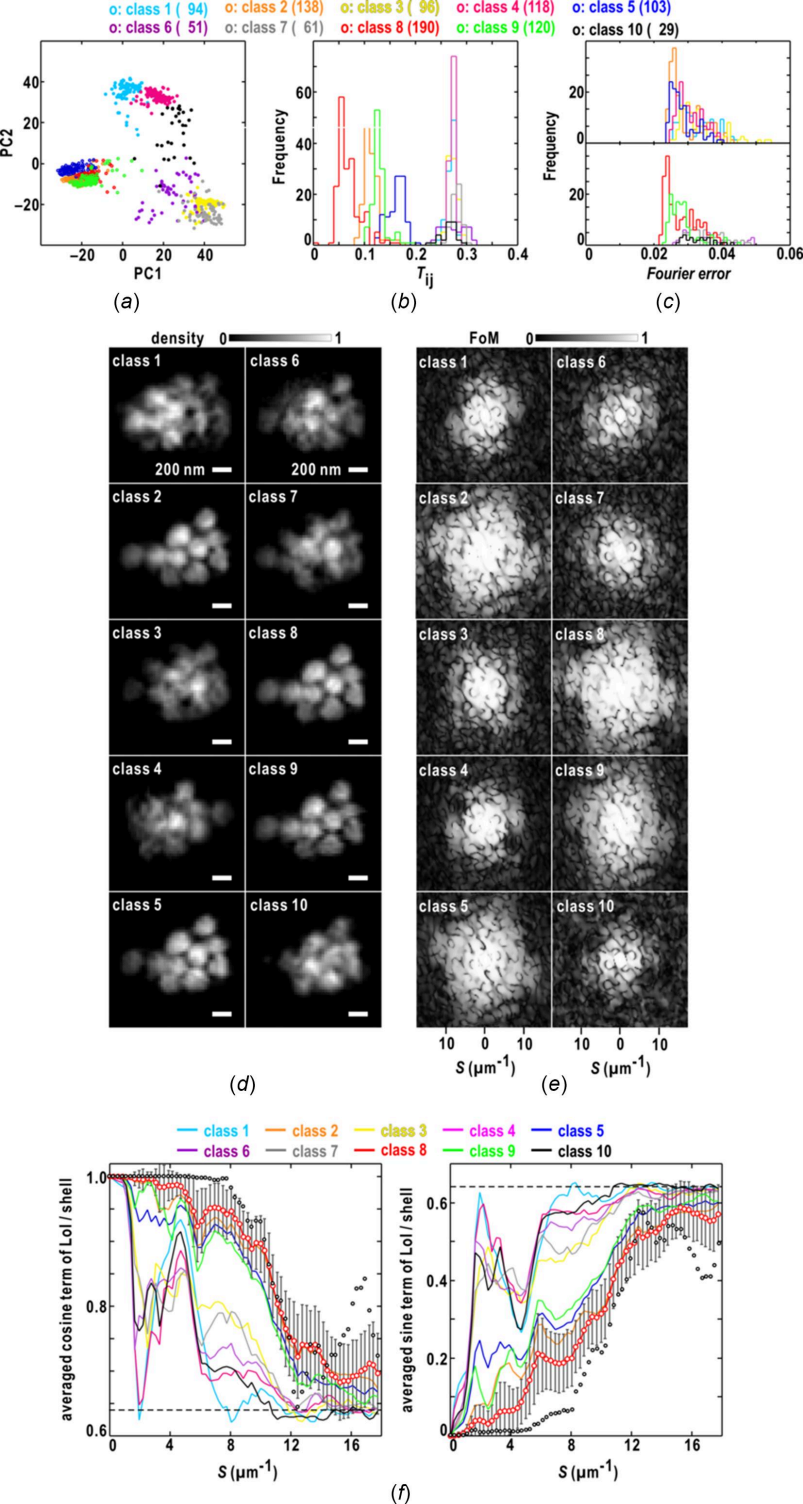
Characterization of the 1000 maps retrieved from the single-shot diffraction pattern in Fig. 1 ▸(*a*). (*a*) Distribution of the 1000 maps divided into ten classes on the plane spanned by the first and second PC vectors. The symbols indicate the positions of the maps on the plane and are colored according to the scheme at the top of the panel. The coloring scheme for the ten classes is used throughout the panels. The number of the map in each class is shown in parentheses. Frequency distributions of the similarity score (*b*) and the Fourier error (*c*) of the maps in each class. (*d*) Comparison of class-representative maps displaying the smallest similarity score against the reference in each class. The densities are illustrated according to the bar at the top of the panel. (*e*) FoMs of structure factors in the ten classes up to a resolution of 18 µm^−1^. The FoM values are shown according to the bar at the top of the panel. (*f*) Resolution-dependences of the averaged cosine (left panel) and sine (right) terms in equation (7) in each class. The curves of class 8 of the two terms are depicted using red open circles and error bars for the standard deviations of the two terms. The open black circles are the two terms for only the pair of maps yielding the smallest similarity scores among the 1000 maps. The dashed line indicates the values of the two terms for the random phase limit [equation (8)].

One thousand maps were retrieved from the diffraction pattern of an aggregate of ten colloidal gold particles in Fig. 1 ▸(*a*), and were divided into ten classes on the plane spanned by the first and second PC vectors [Fig. 3 ▸(*a*)]. The maps in classes 2, 5, 8 and 9 forming a cluster on the plane displayed similarity scores smaller than 0.19 against the reference map, which was one of the pair yielding the smallest similarity score among all the maps [Fig. 3 ▸(*b*)].

Here, we define a quantity in reciprocal space designated the Fourier error of a retrieved map as

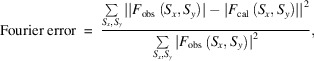

where *F*_cal_(*S*_*x*_, *S*_*y*_) is the structure factor of a retrieved map masked by the support. Because this quantity is related to the target function of the phase retrieval to be reduced, we examined whether this quantity for each map was useful for extracting realistic maps. In this case, as the frequency distributions of the Fourier-error values of the ten classes were heavily overlapped [Fig. 3 ▸(*c*)], we abandoned the use of this quantity for extracting maps of classes 2, 5, 8 and 9 [see Fig. 3 ▸(*d*)].

Fig. 3 ▸(*d*) depicts the map in each of the ten classes, yielding the smallest similarity score against the reference map. In the maps of classes 2, 5, 8 and 9, the ten colloidal gold particles appeared with clear borders and were separated from each other, indicating that the maps in the four classes were successfully retrieved. Among the four correct classes, the maps of class 8 yielded the smallest similarity scores [Fig. 3 ▸(*b*)]. The PR calculations for the other six classes failed, as judged by the unclear and blurred electron densities of the particles. These results imply that the similarity score is a good measure for screening maps in real space. As indicated by the similarity scores, the probable maps were similar to each other, whereas non-realistic maps were different from each other [Fig. 3 ▸(*b*)].

The similarity of the maps in real space was evaluated using the FoM [Fig. 3 ▸(*e*)] and LoI [Fig. 3 ▸(*f*)] in reciprocal space. In the four successfully retrieved classes, particularly classes 2 and 8, the reciprocal regions with FoM values greater than 0.5 extended to a resolution of 18 µm^−1^. In contrast, the FoM values of the non-realistic classes were smaller than 0.5, with a resolution lower than 10 µm^−1^.

Fig. 3 ▸(*f*) shows the resolution-dependent variations of the cosine and sine terms of equation (7). For the pair of maps yielding the smallest similarity score among the 1000 phase-retrieved maps, the phase differences gradually approached the random phase limit [equation (8)] beyond a resolution of 16 µm^−1^. In the realistic classes, the phase differences likely varied in the range 20–30° among the maps up to a resolution of 8 µm^−1^. In the non-realistic classes, judging from the values close to the random phase limit of 0.64 [see equation (8)], the phase differences became random even at a resolution of approximately 2 µm^−1^; this might be a major cause of blurry densities and unclear edges of the averaged maps. It should be noted that there is a boundary of the data-missing area due to the beamstop around the resolution of approximately 2 µm^−1^ [Fig. 1 ▸(*a*)].

**Figure 4:**
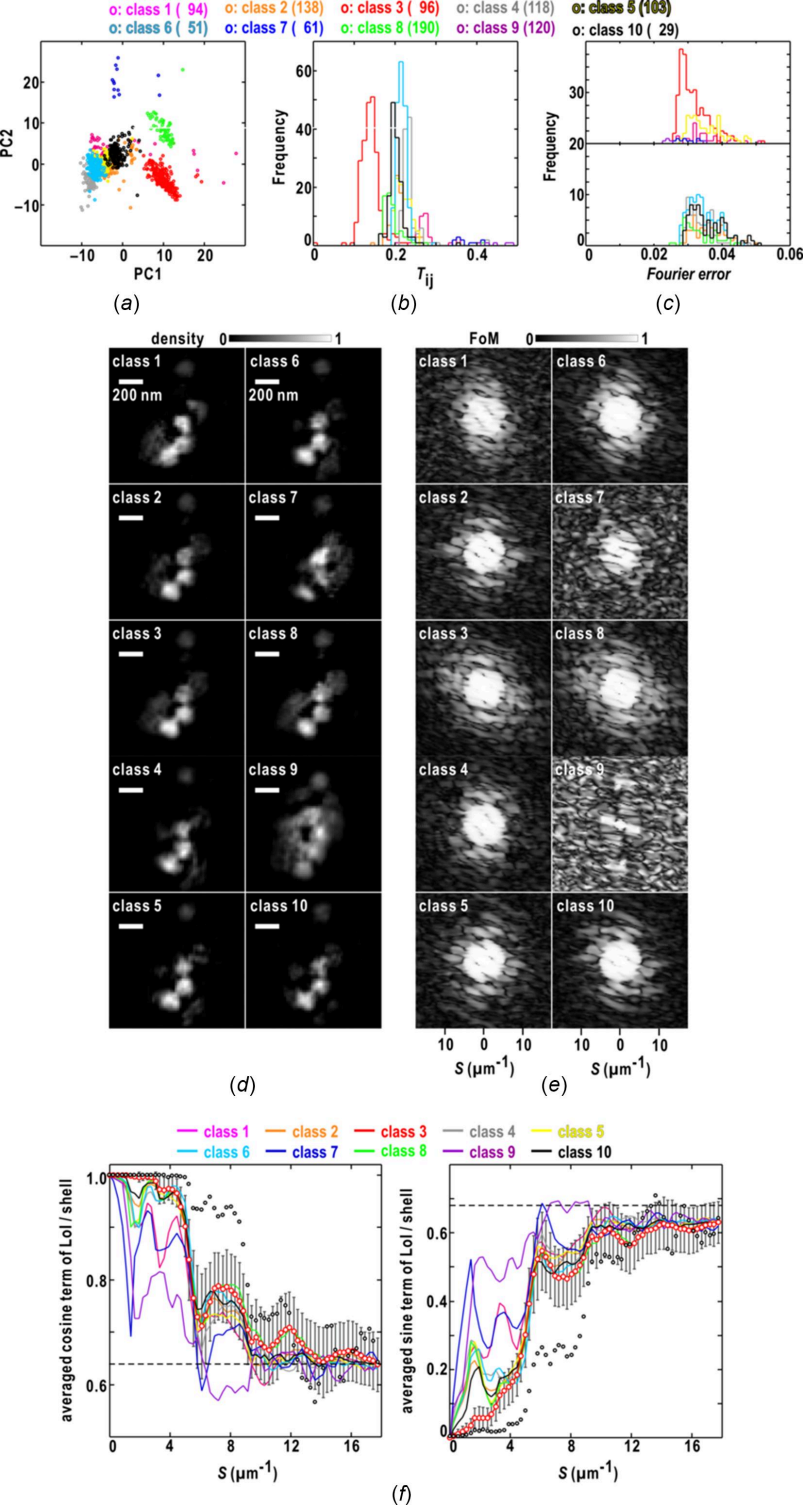
Characterization of the 1000 maps retrieved from the single-shot diffraction pattern in Fig. 1 ▸(*c*). Panels (*a*)–(*f*) are illustrated as in Fig. 3 ▸. In panel (*f*), showing the resolution-dependences of the averaged cosine (left panel) and sine (right) terms in equation (7), the curves of class 3 of the averaged terms are depicted using red open circles and error bars for the standard deviations of the two terms.

Next, we assessed the similarity score for the maps retrieved from another single-shot diffraction pattern shown in Fig. 1 ▸(*c*). Among the ten classes of the 1000 retrieved maps [Fig. 4 ▸(*a*)], only the maps in class 3 showed similarity scores smaller than 0.18 against the reference map [Fig. 4 ▸(*b*)]. In this case, the frequency distribution of the Fourier-error values for maps in class 3 had a peak at 0.026 [Fig. 4 ▸(*c*)], but, in contrast to the similarity score [Fig. 4 ▸(*b*)], the overlap of the frequency distributions among the ten classes made it difficult to extract most of the maps in class 3. Fig. 4 ▸(*d*) depicts the map in each of the ten classes displaying the smallest similarity score against the reference. In the class 3 map [Fig. 4 ▸(*d*)], the three major and three minor densities of colloidal particles were clear, and the minor density at the upper edge was interpreted as the particle being located in the foot part of the XFEL pulse. In the maps of classes 1, 7 and 9, which yielded similarity scores greater than 0.2, the edges of the three major particles were unclear and blurred, indicating failures of the PR calculations. Regarding the FoM of the structure factors of the maps in each class [Fig. 4 ▸(*e*)], classes 3 and 8 maintained FoM values greater than 0.5 beyond a resolution of 10 µm^−1^. In classes 2, 5, 6 and 10, the FoM values were direction-dependent, suggesting that the maps in the four classes had anisotropic similarity.

As shown in Fig. 4 ▸(*f*), the phase differences between the pair of maps yielding the best similarity score approached the random phase limit [equation (8)] beyond a resolution of 14 µm^−1^. In contrast, the maps in each of classes 2, 4, 5, 6, 8 and 10 displayed sudden changes in the cosine and sine terms in the resolution range 1–2 µm^−1^, around the boundary of the data-missing area due to the beamstop [Fig. 1 ▸(*c*)]. The two terms for the maps in each of classes 1, 7 and 9 indicated greater phase differences of structure factors up to a resolution of 17 µm^−1^.

Based on the results, we concluded that the similarity score is good for screening maps phase-retrieved from single-shot diffraction patterns. The similarity score in real space was more convenient for automatically screening maps than the FoM and the two trigonometric terms in reciprocal space [equation (7)]. The trigonometric terms in reciprocal space are important to explain how the similarity score works.

### 4.2. Application to diffraction patterns recorded using synchrotron X-rays

In this section, we apply the similarity score to the maps retrieved from the diffraction patterns of clusters composed of several colloidal gold particles recorded in the SR-XDI experiments. In the SR-XDI experiment, the collection of diffraction patterns from specimens with sizes of several micrometres is possible owing to the wider spatially coherent irradiation area of approximately 10 µm diameter and the smaller beamstop covering a much smaller diffraction angle than those in XFEL-XDI (Takayama *et al.*, 2018 ▸; Kobayashi *et al.*, 2018*b* ▸). In addition, good signal-to-noise ratios in high-diffraction-angle regions might be expected owing to both the relatively large scattering cross-section of specimens with sizes of several micrometres and the exposure time.

**Figure 5:**
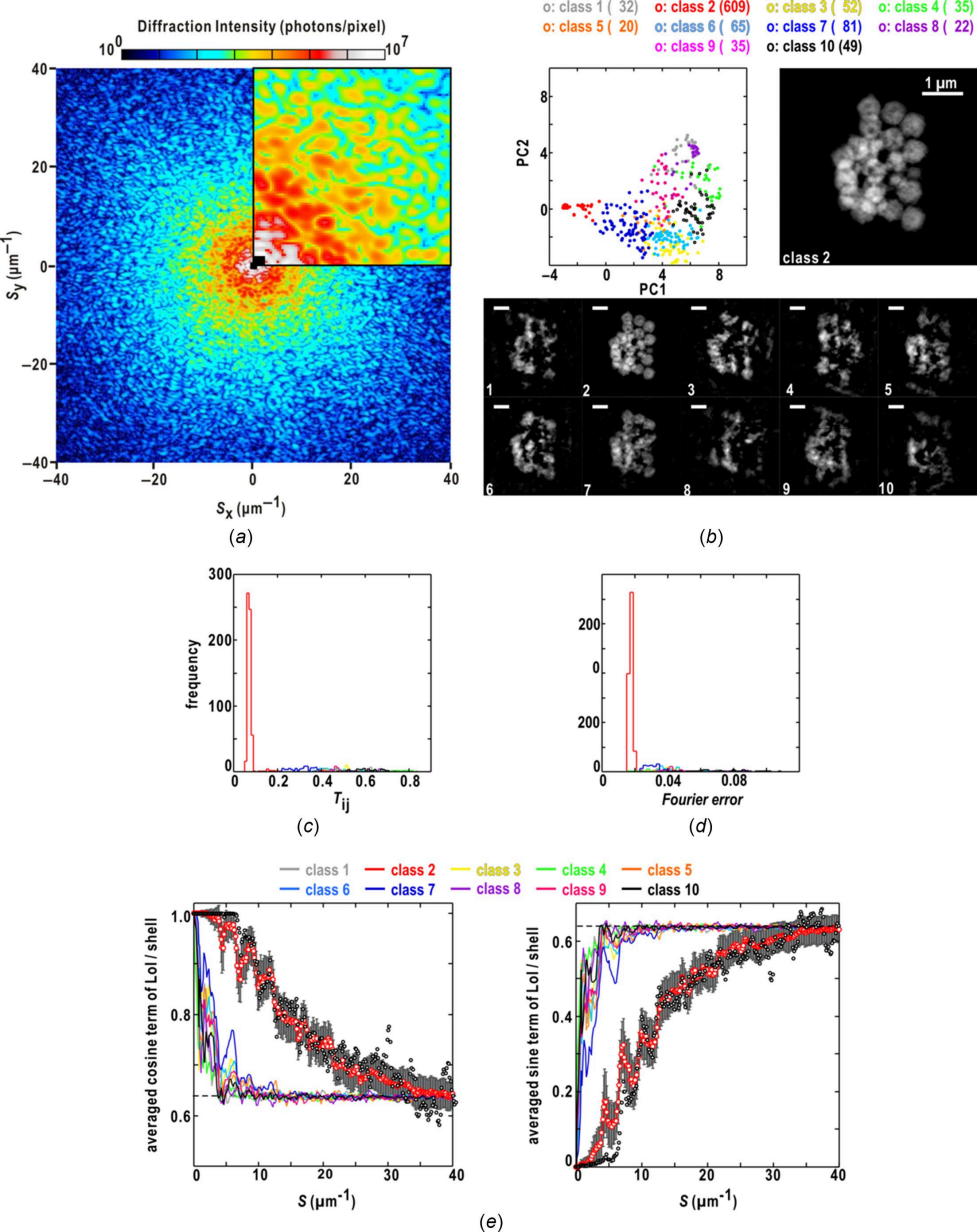
Characterization of the 1000 maps retrieved from the diffraction pattern of an aggregate of more than 20 colloidal gold particles of 400 nm diameter recorded in the SR-XDI experiment. (*a*) Diffraction pattern from the aggregate. The upper-right quadrant is a magnified view of the small-angle region up to 10 µm^−1^. (*b*) Distribution of the retrieved maps in the ten classes on the plane spanned by the first and second PC vectors. The symbols indicate the positions of the maps on the plane and are colored according to the scheme at the top of the panel. The coloring scheme for the ten classes are used throughout the panels. The number of the maps in each class are shown in the parentheses. The enlarged map at the top right is the reference map, which is one of a pair yielding the smallest similarity score among all the maps. The bottom panel compares the class-representative maps displaying the smallest similarity score against the reference in each class. Frequency distributions of the similarity score (*c*) and the Fourier error (*d*) of the maps in each class. (*e*) Resolution-dependences of the averaged cosine (left) and sine (right) terms of equation (7) in each class. The curves of class 2 of the two terms are depicted using red open circles and error bars for the standard deviations of the two terms. The open circles are the two terms for the pair of maps yielding the smallest similarity scores among the 1000 maps. The dashed line indicates the values of the two terms for the random phase limit [equation (8)].

In Fig. 5 ▸, we summarize the application of the similarity score for the maps retrieved from the diffraction pattern of an aggregate composed of more than 20 colloidal gold particles of 400 nm diameter. Of the ten classes divided from the 1000 retrieved maps, only the maps of class 2 were localized in the plane spanned by the two PC vectors [Fig. 5 ▸(*b*)]. Fig. 5 ▸(*b*) depicts the map in each of the ten classes, yielding the smallest similarity score against the reference map. Only the maps of class 2 were realistic, as judged from the clear borders and void-like fine structures of the particles, and yielded a similarity score smaller than 0.08 against the reference [Fig. 5 ▸(*c*)]. As the Fourier-error values of the maps in class 2 displayed a single peak in the frequency distribution [Fig. 5 ▸(*d*)] and correlated with the similarity score values, the Fourier error may be used to extract the class 2 maps in this case.

Fig. 5 ▸(*e*) shows the resolution-dependent variations of the averaged cosine and sine terms of equation (7). For a pair of maps yielding the smallest similarity score, the phase differences in the structure factors of the maps gradually approached the random phase limit beyond a resolution of 30 µm^−1^ [equation (8)]. The two terms of class 2 also asymptotically reached this limit beyond 30 µm^−1^, while those of the other non-realistic classes reached the value within 5 µm^−1^.

**Figure 6:**
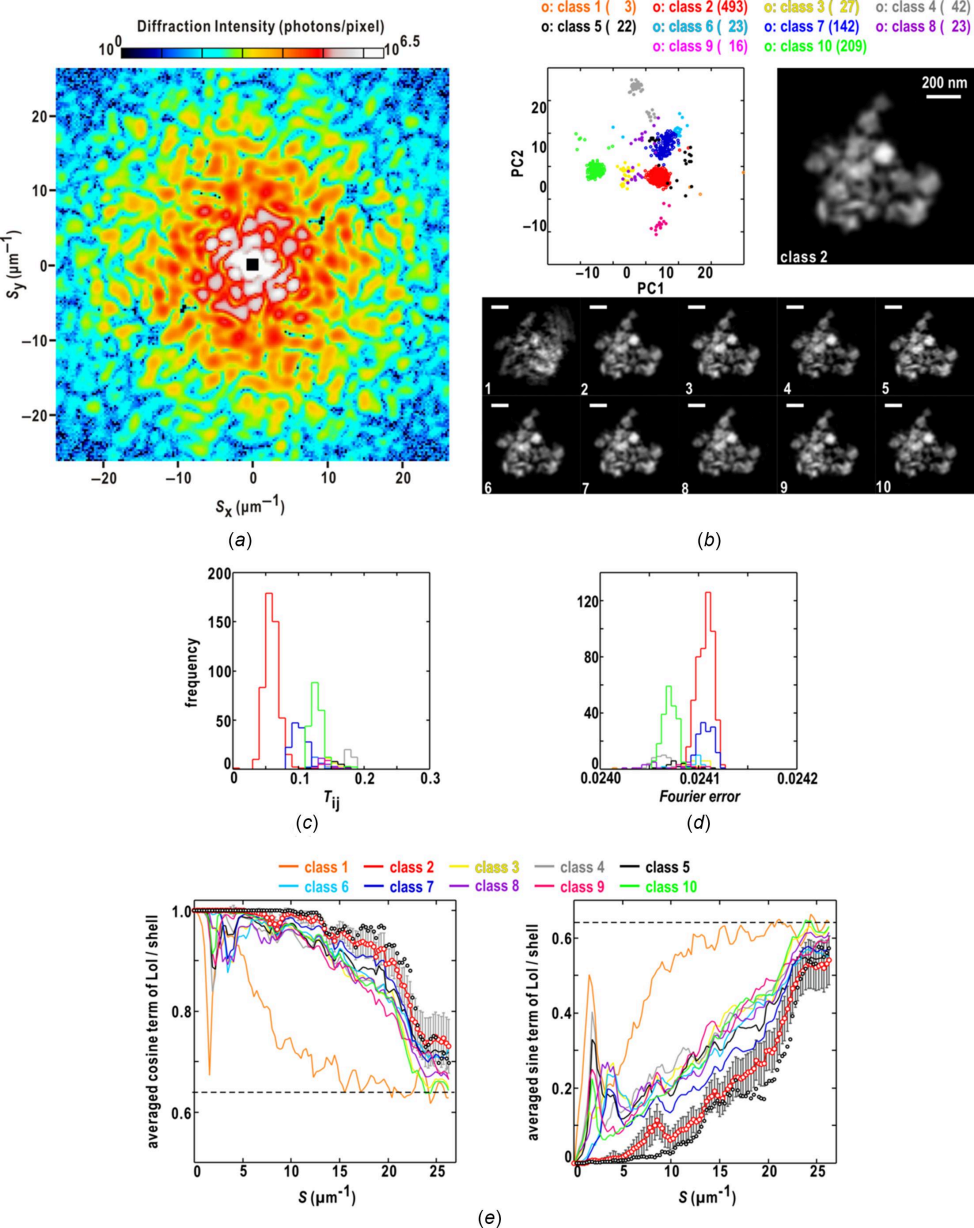
Characterization of the 1000 maps retrieved from the diffraction pattern of a cluster of gold urchin particles recorded in the SR-XDI experiment. All panels are illustrated as in Fig. 5 ▸. The retrieved map at the top right in panel (*b*) is enlarged to clearly show the spikes of the particles. In panel (*e*), showing the resolution-dependences of the averaged cosine (left) and sine (right) terms in equation (7), the curves of class 2 of the two terms are depicted using red open circles and error bars for the standard deviations of the two terms.

As a second example, we assessed the similarity score for maps retrieved from the diffraction pattern of an aggregate of gold urchin particles with irregular shapes (Fig. 6 ▸). As the diffraction pattern included a part of the central speckle in the very small angle region and was recorded with good signal-to-noise ratios up to a resolution of 25 µm^−1^ [Fig. 6 ▸(*a*)], almost all of the maps were successfully retrieved [Fig. 6 ▸(*b*)]. Although the correct maps displayed similarity scores smaller than 0.19, the maps were divided into classes 2–10, probably because of the small differences in the local fine structures, such as convections and protrusions on the surfaces [Figs. 6 ▸(*b*) and 6 ▸(*c*)]. All of the class 2 maps yielded similarity scores smaller than 0.09 against the reference. The Fourier error values of all maps were approximately 0.0241. As the values of the non-realistic maps of class 1 overlapped with the frequency distributions of the other class, it was difficult to exclude the Fourier errors in the maps of class 1 [Fig. 6 ▸(*d*)].

In Fig. 6 ▸(*e*), for the two maps yielding the best similarity score, the phase differences of the structure factors were still non-random beyond a resolution of 25 µm^−1^. For only the maps of class 2, the resolution-dependent variations of the cosine and sine terms of equation (7) were similar to those of the best pair and displayed no sudden changes up to a resolution of 5 µm^−1^. Beyond 23 µm^−1^, the two terms reached the random phase limit of 0.64 [equation (8)], probably because the phase differences among the maps became random.

From the structural analyses of the two diffraction patterns in the SR-XDI experiments, the similarity score in real space acts as a good measure in screening retrieved maps, and the trigonometric terms of equation (7) in reciprocal space were helpful for understanding the correlation between the similarity score and the validity of the maps.

## 5. Discussion

In this study, we characterized the similarity score in correlation with the phase differences in the structure factors of the retrieved maps. Through application to structural analyses for experimental diffraction patterns, we demonstrated the usefulness of the similarity score for screening phase-retrieved maps. Here, we discuss the weak point in the use of the similarity score, the estimation of the effective resolution of the retrieved maps using the score, and the utilization of the score for efficiently performing PR calculations.

### 5.1. Selection of maps using the similarity score

In this study, for selecting realistic maps, which approximate the shape, size and internal structure of a particle, we use the similarity score under the assumption that realistic maps yield the smallest similarity score among the retrieved maps. However, when pairs of non-realistic maps different from the structures of particles are quite similar, this assumption cannot be made. In our experience in structure analyses of aggregates of colloidal gold particles, the breakdown of the assumption occurs at a probability smaller than 0.1%. However, cross-validation using known structural information, for instance from electron microscopy (EM) and fluorescence microscopy, is still necessary for the selected maps, as carried out in our previous works (Kobayashi *et al.*, 2014 ▸; Takayama *et al.*, 2015*a* ▸; Oroguchi *et al.*, 2018 ▸; Kobayashi *et al.*, 2021 ▸).

In this regard, we describe two examples on cross-validation in the structure determination of protein molecules. Even in the estimation of phase set using the multiple heavy-atom replacement method in protein crystallography, we validated the heavy-atom positions in the unit cell of derivative crystals using the phase set in the centric zone of the Bragg reflections obtained from cryogenic TEM (Toyoshima *et al.*, 2000 ▸).

In small-angle X-ray scattering for proteins in solution, the molecular shapes of protein molecules are predicted using an *ab initio* algorithm (Svergun *et al.*, 2001 ▸). As the algorithm is applied to a one-dimensional scattering profile under restraints with respect to the arrangement of small spheres approximating the molecular shape, both realistic and non-realistic models appear in more than 500 trial calculations. Then, after classification of the models using multivariate analysis (Oide *et al.*, 2018 ▸), cross-validation using structural information from other imaging techniques is indispensable for finding realistic molecular structures (Oide *et al.*, 2020 ▸; Oide & Nakasako, 2021 ▸).

The similarity score may be a useful tool for screening realistic maps from a number of trial calculations. However, as we have no experimentally determined phase data in XDI, we keep in mind the possibility of the breakdown of the assumption. Therefore, even for maps yielding small similarity scores, validation for the shape, size and internal structure of the particle is always necessary.

### 5.2. Similarity score and differences in structure factors of maps at low resolution

In the four examples of structure analyses (Figs. 3 ▸–6 ▸ ▸ ▸), the frequency distributions of the similarity scores of phase-retrieved maps in each class are likely correlated with the resolution-dependent variations of the cosine and sine terms for the phase differences of the structure factors between the maps. When the similarity scores in a class are distributed in a range smaller than 0.2, the cosine term is close to 1 and the sine term is near 0 in the small-angle region [Figs. 3 ▸(*f*), 4 ▸(*f*), 5 ▸(*e*) and 6(*e*)]. Owing to the structure amplitudes of the small-angle region, which are much greater than those of the high-angle region [Figs. 1 ▸(*a*), 1 ▸(*c*), 5 ▸(*a*) and 6 ▸(*a*)], when the phase differences of the structure factors of maps are small in the small-angle region, the contribution of the structure factors to the numerator of equation (6) is reduced. Therefore, the small value of the similarity score is interpreted as the retrieved maps viewed at low resolution being similar to each other.

As the contribution of fine structures to the structure factors are dominant at high resolution, the screening of retrieved maps using the similarity score predominantly focuses on the low-resolution structures of the retrieved maps. At low resolution, the FoM takes values close to 1 [Figs. 3 ▸(*e*) and 4 ▸(*e*)], as expected from equation (9). Therefore, the similarity score may be useful in the preliminary screening of realistic maps before applying, for instance, the unweighted pair group method with arithmetic mean analysis, which is the distance-matrix method and employs sequential clustering to yield a rooted phylogenetic tree of image data as used in XFEL-XDI structure analysis (Ekeberg *et al.*, 2015 ▸).

### 5.3. Effective resolution estimated using the similarity score

**Figure 7:**
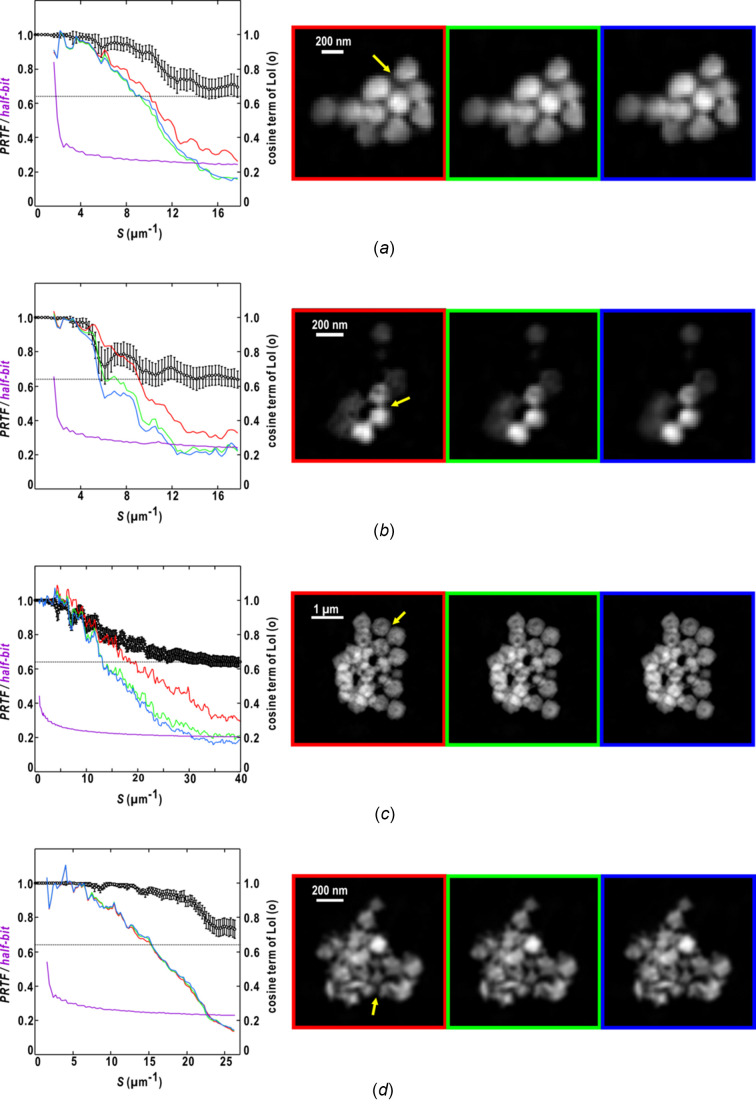
Resolution dependencies of the cosine term of equation (7) (open circles with standard deviations), the PRTF curves (red, green and blue) and the half-bit curves (purple). The cosine terms in panels (*a*)–(*d*) are taken from Figs. 3 ▸–6 ▸ ▸ ▸, respectively. The PRTF curves colored in red, green and blue were calculated for the 2, 10 and 49 smallest similarity scores including the reference, respectively. In the plots, the dotted line indicates the random phase limit [equation (8)]. The maps depicted in the right panel are averaged for the 2 (indicated by red box), 10 (green) and 50 maps (blue) used in the PRTF calculations. The arrows in the maps indicate the small gaps between particles in real space referenced to estimate the effective resolution using the cosine terms and the PRTF curves in reciprocal space.

As the similarity score is dominated by the phase differences between structure factors of retrieved maps in the small-angle region, we discuss how the effective resolution for a set of retrieved maps is quantitatively estimated using the resolution-dependent variation of the cosine (or sine) term in equation (7). Then, we referred to the fine structures of the averaged maps, such as resolved narrow gaps between colloidal particles (arrows in the averaged map in Fig. 7 ▸ and Table 1 ▸). As the phase differences in a resolution shell between two maps are random, the cosine (or sine) term approaches the random phase limit (0.64) [equation (8), Figs. 3 ▸(*f*), 4 ▸(*f*), 5 ▸(*e*) and 6 ▸(*e*)], we adopted the reciprocal of the scattering vector length, where the cosine curve for sets of retrieved maps approaches the random-phase limit, as an effective resolution of the averaged map.

As a result, the effective resolution estimated in each of Figs. 7 ▸(*b*) and 7 ▸(*c*) was approximately twice the gap dimensions. From the point of view of the resolution in microscopy that is given by the Fourier component with the shortest period, half of the minimum period of the Fourier component is resolved. In Figs. 7 ▸(*a*) and 7 ▸(*d*), as the cosine terms were still greater than 0.64 at the highest resolution recorded, the effective resolution of the averaged map was assumed to be the highest resolution of the diffraction pattern used in the PR calculation (Table 1 ▸).

We also estimated the effective resolution using the PRTF curves [equation (10)]. The curves were calculated independently for ten and 50 maps from the best classes in each structure analysis and additionally for two maps yielding the smallest similarity score as a reference (Fig. 7 ▸). For maps displaying small variations in electron density, the greater number of maps used in the PRTF calculation likely correlated with the more rapid decrease of the curves [Figs. 7 ▸(*a*)–7(*c*)]. In contrast, for the maps that were very similar to each other, the PRTF curves were almost independent of the number of maps [Fig. 7 ▸(*d*)]. A comparison of the four cases indicated that the PRTF curves were likely advantageous for monitoring the degree of the convergence of the maps.

When the threshold for estimating the effective resolution of the maps from the PRTF curve was set to 0.5 or 1/*e* (∼0.37) (Chapman *et al.*, 2006 ▸), the estimated effective resolution was lower than that to resolve the narrow gaps between particles. Instead, we tried to use the half-bit information threshold curve (van Heel & Schatz, 2005 ▸), which was proposed in conjunction with the Fourier shell correlation (FSC) curve (Rosenthal & Henderson, 2003 ▸) for estimating the effective resolution in single particle analysis using TEM. Although the definition of the FSC curve is different from that of the PRTF curve, both curves essentially measure the distribution of phase values. The half-bit curve, which is correlated with the information content in voxels (pixels) in a resolution shell, is known to allow a reliable interpretation of the resolution level in TEM analysis (van Heel & Schatz, 2005 ▸). The effective resolution estimated from the scattering vector length, where the PRTF curve crossed the half-bit curve, was comparable with the doubled gap dimension between colloidal particles (Fig. 7 ▸ and Table 1 ▸).

As a result, the effective resolution estimated using the cosine term and the random-phase limit of 0.64 was comparable with that using the PRTF and half-bit curves. The estimation of the effective resolution of the retrieved maps is still under debate – further experiences regarding the structure analyses of various aggregates of metal particles are necessary.

### 5.4. Similarity score and landscape on the distribution of retrieved maps

**Figure 8:**
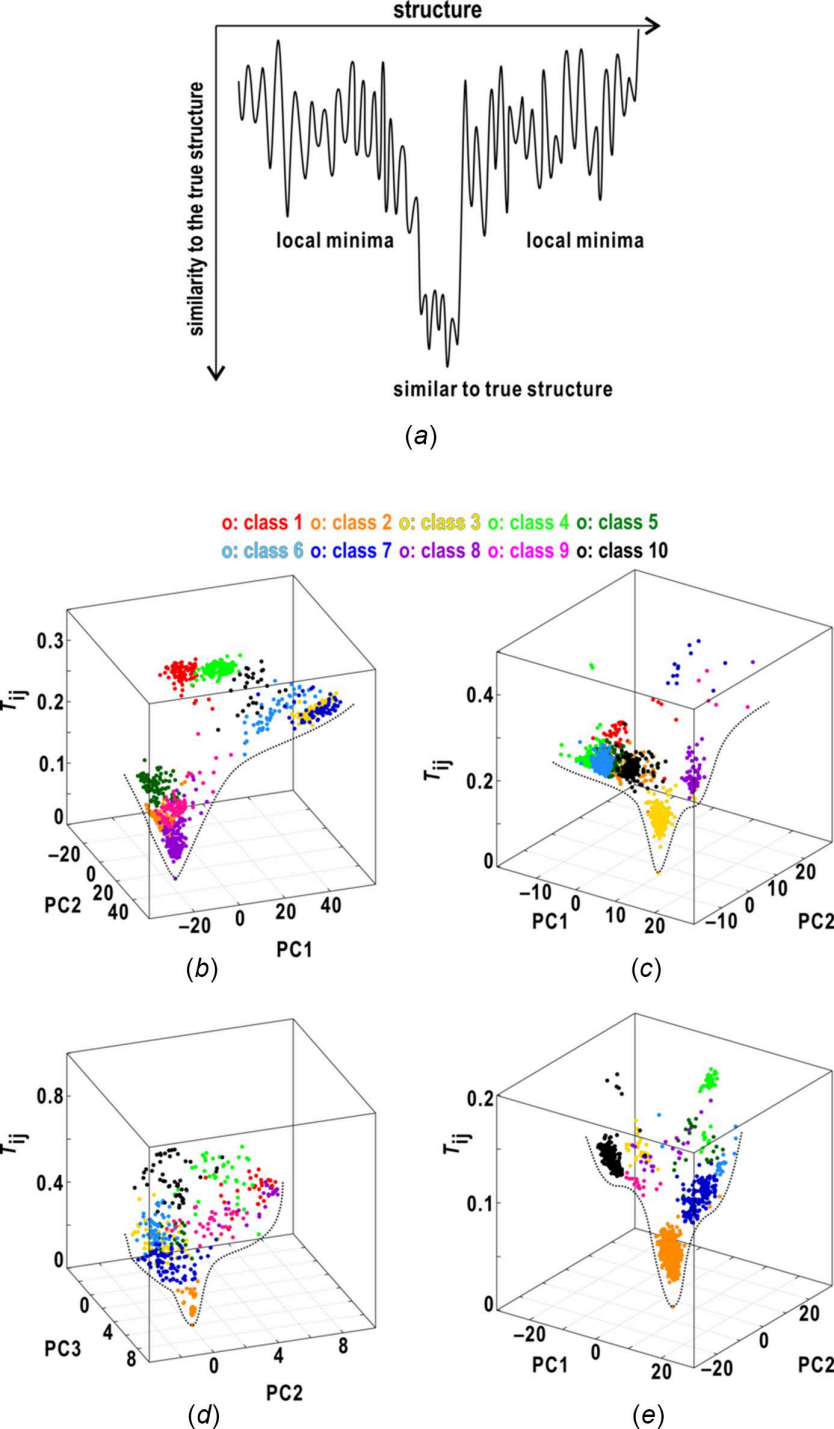
(*a*) Schematic illustration of the landscape in the multidimensional space spanned by the similarity score and structure axes. Panels (*b*)–(*e*) show the distributions of the retrieved maps taken from Figs. 3 ▸–6 ▸ ▸ ▸, respectively, in the three-dimensional space spanned by the similarity score and two PC vectors representing the variations of electron densities. The dots indicate the positions of the maps in the space, and are colored according to the scheme at the top of panels (*b*) and (*c*). In each panel, the dotted lines are the rough borders of the distributions of the maps.

In the previous study, we assumed that the phase-retrieved maps were distributed on a funnel-shaped landscape in multi-dimensional space spanned by the similarity score and pixel values of the maps, reflecting the structure, as schematically illustrated in Fig. 8 ▸(*a*) (Sekiguchi *et al.*, 2017 ▸). Based on this idea, the retrieved maps in Figs. 3 ▸–6 ▸ ▸ ▸ were plotted in the three-dimensional space spanned by the similarity score and the two PC vectors representing the major characteristics of the electron density distributions in all the retrieved maps [Figs. 8 ▸(*b*)–8(*e*)]. The maps were roughly distributed along funnel-shaped surfaces, and the distributions were different from each other probably due to the area of the missing small-angle regions, the signal-to-noise ratios and the oversampling ratios. The maps displaying small similarity scores were concentrated near the bottom part of the funnel. In contrast, the other maps with large scores were distributed around the upper edges of the funnel [Fig. 8 ▸(*b*)], or in local minima [Fig. 8 ▸(*c*)].

This representation is helpful for understanding characteristics of the funnel-shaped surface. As even maps displaying large scores are located in a local area on the two-dimensional planes, the landscape of the area is expected to be rugged as illustrated in Fig. 8 ▸(*a*). In the case of the retrieved maps of the aggregate of gold urchin particles, the nine classes discretely distributed near the bottom of the funnel [Fig. 8 ▸(*e*)] suggested local and shallow minima even at the bottom of the funnel, probably because of the small differences in the fine structures.

The visualized distributions encouraged us to steer the PR calculations toward the most probable solution for the observed diffraction pattern by referring to the similarity scores during the progress of the calculations. For instance, in parallelly conducted PR calculations, the modification of maps by those near the bottom of the funnel may increase the probability of successful calculations (Yoshida *et al.*, 2024 ▸).

## Figures and Tables

**Table 1:** Effective resolution estimated using the cosine term of equation (7) and the PRTF curves of Fig. 7 ▸ with appropriate threshold values

	Effective resolution estimated (µm^−1^ / nm) (number of maps used)			
Gap between particles (nm)	From cosine term using random phase limit	From PRTF curve and half-bit threshold curve		
Panel (*a*) / 27.8	18.0 / 55.6 (190)	18.0 / 55.6 (2)	16.0 / 62.5 (10)	15.6 / 64.3 (50)
Panel (*b*) / 27.8	14.2 / 70.4 (235)	18.0 / 55.6 (2)	18.0 / 55.6 (10)	18.0 / 55.6 (50)
Panel (*c*) / 12.5	32.6 / 30.6 (609)	40.0 / 25.0 (2)	40.0 / 25.0 (10)	34.5 / 29.0 (50)
Panel (*d*) / 18.5	26.0 / 38.5 (493)	24.6 / 40.6 (2)	24.6 / 40.6 (10)	24.6 / 40.6 (50)
